# Unbiased logic-tree data for earthquake-induced landslide hazard maps for low-to-moderate magnitude events

**DOI:** 10.1016/j.dib.2020.105940

**Published:** 2020-06-27

**Authors:** Martín J. Rodríguez-Peces, José C. Román-Herrera, José A. Peláez, José Delgado, Meaza Tsige, Salvatore Martino, Jesús Garrido

**Affiliations:** a Department of Geodynamics, Stratigraphy and Paleontology, Faculty of Geological Sciences, University Complutense of Madrid, C/José Antonio Novais, 12, 28040 Madrid, Spain; b Department of Physics, University of Jaén, Campus Las Lagunillas, Jaén 23071, Spain; c Department of Environment and Earth Sciences, University of Alicante, Ap. Correos 99, 03080 Alicante, Spain; d Department of Earth Sciences and Research Center for Geological Risk (CERI), University of Roma “Sapienza”, P.le A.Moro, 5, 00185, Italy; e Department of Civil Engineering, University of Granada, Campus Fuentenueva, Av. Severo Ochoa s/n, 18071 Granada, Spain

**Keywords:** Landslide, Earthquake, Seismically-induced landslide, Hazard map, Logic-tree

## Abstract

Land-use planning in regard of earthquake-triggered landslides is usually implemented by means of the production of hazard maps. The well-known Newmark rigid block methodology is the most frequent used approach for this purpose. In this method, slope stability is evaluated by the estimation of the Newmark displacement, which is used to set different categories of hazard. This methodology presents limitations due to the difficulty of incorporating the variability of the used variables. For that reason, the logic-tree approach has been used in order to incorporate the epistemic uncertainties and compute probabilistic seismic-landslide hazard maps. However, the used weights in the logic-tree are usually set for each branch based on an expert judgement or subjective criteria. This article provide data obtained from the use of logic-tree methodology; this dataset is useful for deriving the unbiased weights to use in such methodology and in moderate-to-low magnitude scenarios. The data presented here are related to the article entitled “Obtaining suitable logic-tree weights for probabilistic earthquake-induced landslide hazard analyses” (Rodríguez-Peces et al., 2020) [1].

Specifications Table

**Table:** 

**Subject**	Earth and Planetary Sciences
**Specific subject area**	Engineering Geology, Seismically-induced landslides
**Type of data**	Table (Excel file)
**How data were acquired**	Following a logic-tree schema, a set of values representative of variables that participate in the evaluation of Newmark displacement were chosen (geotechnical, size-depth to failure surface, ground motion evaluation and regression models for estimating displacements). Displacements were computed according to Newmark rigid-body methodology using a new software code [2] written in Phyton 2.7 and ArcGIS© 10 software.
**Data format**	Table with raw and analyzed data.
**Parameters for data collection**	For each combination of input parameters, the surface of territory characterized by a minimum slope of 10° and Newmark displacement of 1 cm was computed (%TAC). Percentage of landslides triggered by the reference event (2011, Mw 5.1, Lorca earthquake) located on pixels with displacements above 1 cm was also computed (or instabilities correctly identified,%GFC). Additionally, only models with a success rate (%SR) above 1% were used.
**Description of data collection**	Dataset comprises several columns depicting the value of input parameters (percentile of each input, value of depth to failure surface and regression model used for computing Newmark displacements) and additional columns showing%GFC and%TAC.
**Data source location**	City/Town/Region: Lorca, Murcia
	Country: Spain
**Data accessibility**	With the article
**Related research article**	Author's name: Rodríguez-Peces, M.J.; Román-Herrera, J.C.: Peláez, J.A.; Delgado, J.; Tsige, M.; Missori, C.; Martino, S.; Garrido, J.
	Title: Obtaining suitable logic-tree weights for probabilistic earthquake-induced landslide hazard analyses
	Journal: Engineering Geology

## Value of the Data

• Available methods to obtain logic-tree weights in a probabilistic earthquake-induced landslide hazard analysis are based on an expert judgement or subjective considerations. Data provided in this paper provide a basis for obtaining unbiased logic-tree weights.
• Data are especially valuable for those researchers working in areas of moderate-to-low magnitude earthquakes and interested in evaluating hazard related to seismically-induced landslides.
• Data represent the basis for a research in progress with the aim of producing seismic-induced landslide hazard maps for moderate-low magnitude events along the main lifelines in Southern Spain.
• No raw data of Newmark displacement for preparing probabilistic earthquake-induced landslide hazard results are freely available. Data can be used for estimating new seismic-induced landslide hazard maps for moderate-low magnitude events, or for complementing other author's data.

## 1 Data description

Database consists of a table structured in nine columns, which list the combination of input variables and efficiency parameters for each computed seismically-induced landslide hazard map.

The first five columns comprise the combination of input parameters used for each hazard map: slope instability size (t), specific weight (d), cohesion (c), friction angle (phi) and Newmark displacement empirical relationship (Ec).

The next four columns comprise efficiency parameters: percentage of total area identified as landslides (%TAC =% Total Area Covered), percentage of correctly identified landslide areas (%GFC =% Ground Failure Capture), difference between%GFC and%TAC (%Difference) and success rate percentage (%SR), defined as the multiplication of%GFC and% Difference.

## 2 Experimental design, materials, and methods

**Fig. 1:**
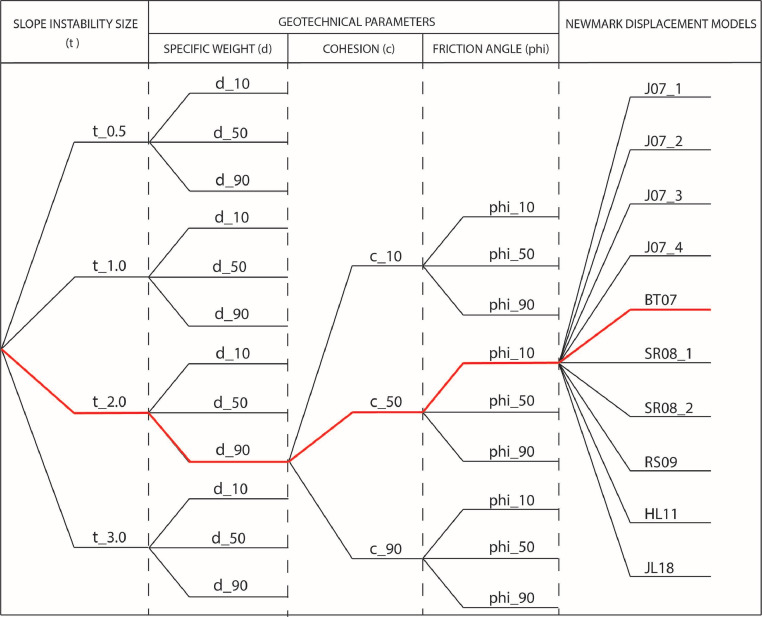
Structure of the logic-tree for a probabilistic seismic landslide hazard mapping assessment. The red line shows the path of an example of hazard map that comprises a landslide size of 2 m, the value at the 90th percentile of the specific weight, the 50th percentile of cohesion and the 10th percentile of the friction angle, and BT07 Newmark displacement equation.

A logic-tree approach is proposed in order to incorporate the variability of variables used in the procedure for computation of seismic-induced landslide hazard maps. The logic-tree has been divided into three sections having regard to the variability of the landslide size (represented by the depth to failure surface), geotechnical parameters (i.e., specific weight, cohesion and friction angle) and Newmark displacement empirical models (Fig. 1). For these models, we have worked with data from the area of Lorca (SE Spain), where a Mw 5.1 earthquake occurred in 2011 triggered several hundreds of landslides [3]. Data include characteristics of landslide triggered (location and size), ground motion severity (peak ground acceleration and Arias intensity) and geotechnical properties of materials affected by landslides induced by this event.

In the first section, the variability of the landslide size was considered according to the slope instability inventory of the 2011, Mw 5.1, Lorca earthquake [3]. The branches of the logic-tree were defined by the values of landslide size (t) of 0.5, 1.0, 2.0 and 3.0 m.

For the second section, different values of specific weight, cohesion and friction angle were set to each geological unit. These parameters were derived from a geotechnical database compiled in the Lorca area. All data has been statistically processed using the IBM SPPS Statistic 25 statistical software. Thereafter, the logic-tree branches for this section of each geotechnical parameter have been chosen as 10, 50 and 90 percentiles.

As for the third section of the logic-tree, eleven models have been selected for estimating Newmark displacement from the large number of available relationships, which can be used in the Lorca case [4], [5], [6], [7], [8]–9].

Finally, a new software code was developed in order to obtain the landslide hazard maps in terms of Newmark displacement considering the different branches of the logic tree [2]. That semiautomatic code was written in Phyton using a geographic information system (ArcGIS© 10 software).

The prediction efficiency of each estimated seismically-induced hazard map was computed based on the success rate percentage (%SR) by combining the efficiency parameters used by McCrink [10]. A higher efficiency could be found maximizing the percentage of correctly identified landslide (%GFC), minimizing the percentage of the total area identified as landslides (%TAC) and maximizing the difference between%GFC and%TAC (%Difference).

## Declaration of Competing Interest

The authors declare that they have no known competing financial interests or personal relationships that have, or could be perceived to have, influenced the work reported in this article.

## Appendix Supplementary materials

Image, application 1
